# The role of antibody expression and their association with bladder cancer recurrence: a single-centre prospective clinical-pilot study in 35 patients

**DOI:** 10.1186/s12894-020-00759-3

**Published:** 2020-11-25

**Authors:** Peter Ella-Tongwiis, Rebecca May Lamb, Alexander Makanga, Iqbal Shergill, Stephen Fôn Hughes

**Affiliations:** 1 North Wales Clinical Research Centre, Betsi Cadwaladr University Health Board (BCUHB), Wrexham Maelor Hospital, Wrexham, Wales UK; 2 Faculty of Social and Life Sciences, Wrexham Glyndwr University, Wrexham, UK; 3 Department of Histopathology, Ysbyty Glan Clwd, Betsi Cadwaladr University Health Board (BCUHB), Wrexham, UK; 4 Department of Biological Sciences, University of Chester, Chester, UK; 5 Department of Urology, BCUHB Wrexham Maelor Hospital, Wrexham, Wales UK

**Keywords:** Immunohistochemistry, Recurrence, Biomarkers, Bladder

## Abstract

**Background:**

Bladder cancer (BC) is the 10th most common cancer in the UK, with about 10,000 new cases annually. About 75–85% of BC are non-muscle invasive (NMIBC), which is associated with high recurrence and progression rates (50–60% within 7–10 years). There are no routine biomarkers currently available for identifying BC patients at increased risk of developing recurrence. The focus of this research study was to evaluate antibody expression in BC patients and their association with cancer recurrence.

**Methods:**

35 patients scheduled for TURBT were recruited after written informed consent. Ethical approval for the project was granted via IRAS (REC4: 14/WA/0033). Following surgical procedure, tissues were preserved in 10% buffered formalin and processed within 24 h in FFPE blocks. 7 sections (4 µm each) were cut from each block and stained for CD31, Human epidermal growth factor receptor-2 (HER-2), S100P, Cyclooxygenase-2 (COX-2), VEGFR-3 thrombomodulin and CEACAM-1 using immunohistochemistry. Clinical outcome measures (obtained via cystoscopy) were monitored for up to 6 months following surgical procedure.

**Results:**

There was significantly increased expression of CD31 (*p* < 0.001), HER-2 (*p* = 0.032), S100P (*p* < 0.001), COX-2 (*p* < 0.001), VEGFR-3 (*p* < 0.001) and decreased expression of thrombomodulin (*p* = 0.010) and CEACAM-1 (*p* < 0.001) in bladder tumours compared to normal bladder tissues. HER-2 expression was also significantly associated with cancer grade (*p* = 0.003), especially between grade 1 and grade 2 (*p* = 0.002) and between grade 1 and grade 3 (*p* = 0.004). There was also a significant association between cancer stage and HER-2 expression (*p* < 0.001). Although recurrence was significantly associated with cancer grade, there was no association with antibody expression.

**Conclusion:**

Findings from the present study may indicate an alternative approach in the monitoring and management of patients with BC. It is proposed that by allowing urological surgeons access to laboratory markers such as HER-2, Thrombomodulin and CD31 (biomarker profile), potentially, in the future, these biomarkers may be used in addition to, or in combination with, currently used scoring systems to predict cancer recurrence. However, verification and validation of these biomarkers are needed using larger cohorts.

## Background

Globally, there were approximately 549,393 new bladder cancer (BC) diagnoses in 2018 of which about 90% are Transitional cell carcinomas (TCC) [1]. An estimated 80% of TCC are Non-Muscle Invasive (NMIBC) while about 20% are Muscle Invasive BC (MIBC). High recurrence rates in NMIBC means patients are exposed to frequent hospital visits with increased risks of infection and post-operative complications. The Transurethral Resection of the Bladder Tumour (TURBT) is still the main procedure for treatment and diagnosis of BC. Insufficient resection has been associated with an increased risk of early recurrence [2].

Although there is intense ongoing BC-related research, no routinely used biomarkers exist for predicting recurrence following TURBT. Cyclooxygenase-2 (COX-2) plays important roles in the inflammatory response [3] with high expression of COX-2 being reported in high grade and high stage colorectal cancer [4] and endometrial carcinoma [5].

Thrombomodulin is a 74 kDa membrane receptor expressed on endothelial cells with important roles in physiological coagulation, inflammation and cancer promotion [6, 7]. The effects of Thrombomodulin activity in cancer have been linked to its roles in anticoagulation, anti-inflammation and tissue adhesion and proliferation [8].

The S100 proteins are associated with cellular processes such as regulation of cell cycle, growth, transcription and differentiation [9]. It is thought that, when secreted, S100P may have paracrine or autocrine signalling effects [10], while cellular expression of S100P may interact with growth factors and receptors leading to several effects such as proliferation [9]. The association between S100P and cellular processes such as cell survival, proliferation, tumour invasion and angiogenesis have been studied by various researchers [9, 11–13].

As an oncogene, HER2 over-amplification causes tumourigenesis due to activation of signalling pathways such as MAPK [14, 15]. HER2 gene amplification and higher protein expression occurs in about 25% of people with metastatic breast cancer [16].

Even though VEGFR-3 expression is usually located on endothelial cells within the lymphatic system [17] and has been mainly involved in lymphangiogensis [18]. Some studies have observed that any inhibition of VEGFR-3 activity, reduces Angiogenic sprouting and vascular network formation [19]. Due to the high recurrence rates in BC, measurement of VEGF and their receptors may provide useful for predicting recurrence and progression.

Decreased expression, down regulation or loss of CEACAM-1 expression in tumour cells has previously been reported [20, 21]. In hepatocellular carcinoma, loss of CEACAM-1 expression is significantly associated with high grade and poor survival [22].

CD31 plays various roles in angiogenesis, leucocyte migration and activation of integrins [23, 24] and may be secreted, membrane bound or localised intracellularly. Increased CD31 expression has also been associated with worst cancer specific survival [25] and poor prognosis [26].

Findings from the present study may indicate an alternative approach in the monitoring and management of patients with BC. It is proposed that by allowing urological surgeons access to laboratory markers such as HER-2, Thrombomodulin and CD31 (biomarker profile), potentially, in the future, these biomarkers may be used in addition to, or in combination with, currently used scoring systems to predict cancer recurrence. However, verification and validation of these biomarkers are needed using larger cohorts.

## Methods

### Ethical approval and subject recruitment

Permission for this research study was granted by the Research Ethics Service (REC reference 14/WA/0033). Subjects recruited for this research attended the Betsi Cadwaladr University Health Board (BCUHB) Wrexham Maelor Hospital, North Wales for elective procedures for the treatment/management of BC (Table 1). 35 patients (average age = 74, males = 29 and females = 6) prospective patients scheduled for a Transurethral Resection of the Bladder tumour (TURBT) for the treatment of BC, were recruited into this research study after informed consent.

**Table 1:** Demographic and clinical characteristics of recruited subjects

Age (years)	
Mean	74
Median	75
Range	43–95
Sex	
Male	29
Female	6
Cancer stage	
pTa	22
pT1	6
pT2	5
Cancer grade	
Grade 1	9
Grade 2	11
Grade 3	13
Cancer recurrence	
Recurrence	14
No recurrence	21

### Tissue preservation, processing and microtomy

Formalin Fixed, Paraffin Embedded (FFPE) tissue blocks of recruited patients were retrieved from storage at the histopathology department of Ysbyty Glan Clwyd, North Wales (UK). At least 12 sections (4 µm each) were cut from each patient tissue block using a microtome, placed on positively charged glass microscope slides and incubated at 60^0^ C for at least 30 min before proceeding with IHC. This process enhances adherence of the tissue to the glass slides and ensures stability of the tissue.

### Antibodies

Monoclonal anti-CEACAM-1/CD66a (clone: 283324; R&D systems UK), monoclonal anti-CD31 (clone: JC70; cell marque UK), Polyclonal anti-COX-2 (Clone: SP21; cell marque UK), monoclonal anti-HER-2/neu (Clone: 4B5; Roche diagnostics UK), monoclonal anti-S100P (clone: 16/f5; Cell marque UK), monoclonal anti-thrombomodulin (clone: 1009; Cell marque UK) and monoclonal anti-VEGFR-3 (clone: AF349; R&D systems UK) were used in this study. Antibodies were stored and treated according to manufacturer’s instructions.

### Immunohistochemistry

Tissue slides were dewaxed using 1X EZ prep solution (Ventana Medical Systems, UK), heat and vortex mixing and antigen retrieval was carried out using cell condition 1. VENTANA Antibody Diluent with Casein was used to block endogenous proteins. This was followed by the application of primary antibodies which primarily were mouse and rabbit IgG. The antigens were then visualised using Ventana Ultraview DAB universal detection kit. The slides were then counter stained using Ventana haermatoxylin 1. The slides were washed in warm soapy water to remove residual liquid coverslip (oil) and the then dehydrated in a series of alcohol and DPX mounted on Dako HE autostainer. For each IHC stain, both positive and negative control tissue were used to ensure validity of the results.

### Assessing recurrence

In all patients recruited into this research study, follow up data immediately available from follow up clinics was retrieved. This information was obtained from outpatient clinic as well as BCUHB clinical portal.

Investigating the long-term overall survival rates, 5-year and 10-year recurrence and progression data are, however, beyond the scope of this research. However, the short-term information available (up to 2 years post TURBT) will be discussed in relation to the biomarkers measured and may ultimately provide sufficient and accurate clinical data that may help predict or forecast NMIBC recurrence and progression.

### Statistical analysis

Statistical analysis was undertaken using SPSS (version 26). With regards to tissue analysis, a chi-square test was performed to determine the difference between antibody staining in bladder tumour cells in comparison with normal urothelium. A Kruskal Wallis test was used to determine the association between clinopathological features (such as cancer grade and stage) and expression. In cases where there was a significant difference, further post-hoc testing was performed using the Mann–Whitney test.

## Results

### CD31 tissue expression in BC patients

**Fig. 1:**
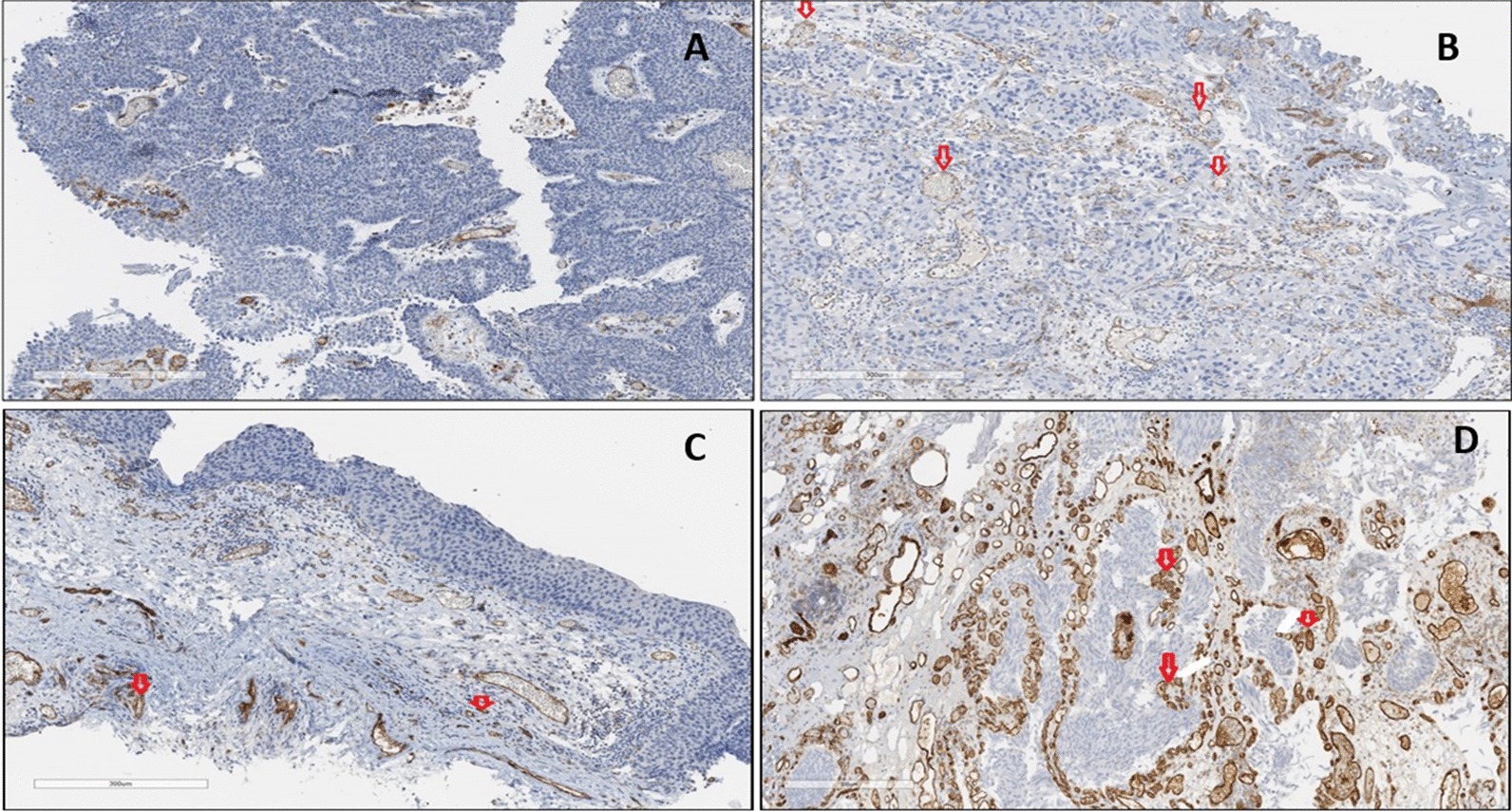
Anti-CD31 antibody staining in BC tissues. **a** Negative: No staining or positive cytoplasmic/membranous staining in < 5% of VE cells in BC tissues. **b** Positive (+: Weak intensity cytoplasmic/membranous staining in up to 20% of VE cells in BC tissues. **c** Positive (++): Moderate intensity cytoplasmic/membranous staining in 20–50% of VE cells in BC tissues. **d** Positive (+++): Strong cytoplasmic/membranous staining in > 50% of VE cells in BC tissues. DAB detection. ×40 magnification. VE, vascular endothelial

Anti-CD31 primary monoclonal antibody demonstrated cytoplasmic and membranous staining patterns in endothelial cells (Fig. 1). Vascular endothelial cells in BC tissues had significantly higher anti CD31 expression, compared to normal underlying tissues (X^2^(3) = 20.353, *p* < 0.001), as determined by the Chi-square test. With respect to cancer grading and staging, there was no significant association between patients’ cancer grade and CD31 (X^2^(2) = 2.719, *p* = 0.257), as determined by the Kruskal–Wallis test. However, there was a significant association with cancer stage (X^2^(2) = 12.276, *p* = 0.002), as determined by the Kruskal–Wallis test.

### Thrombomodulin tissue expression in BC patients

**Fig. 2:**
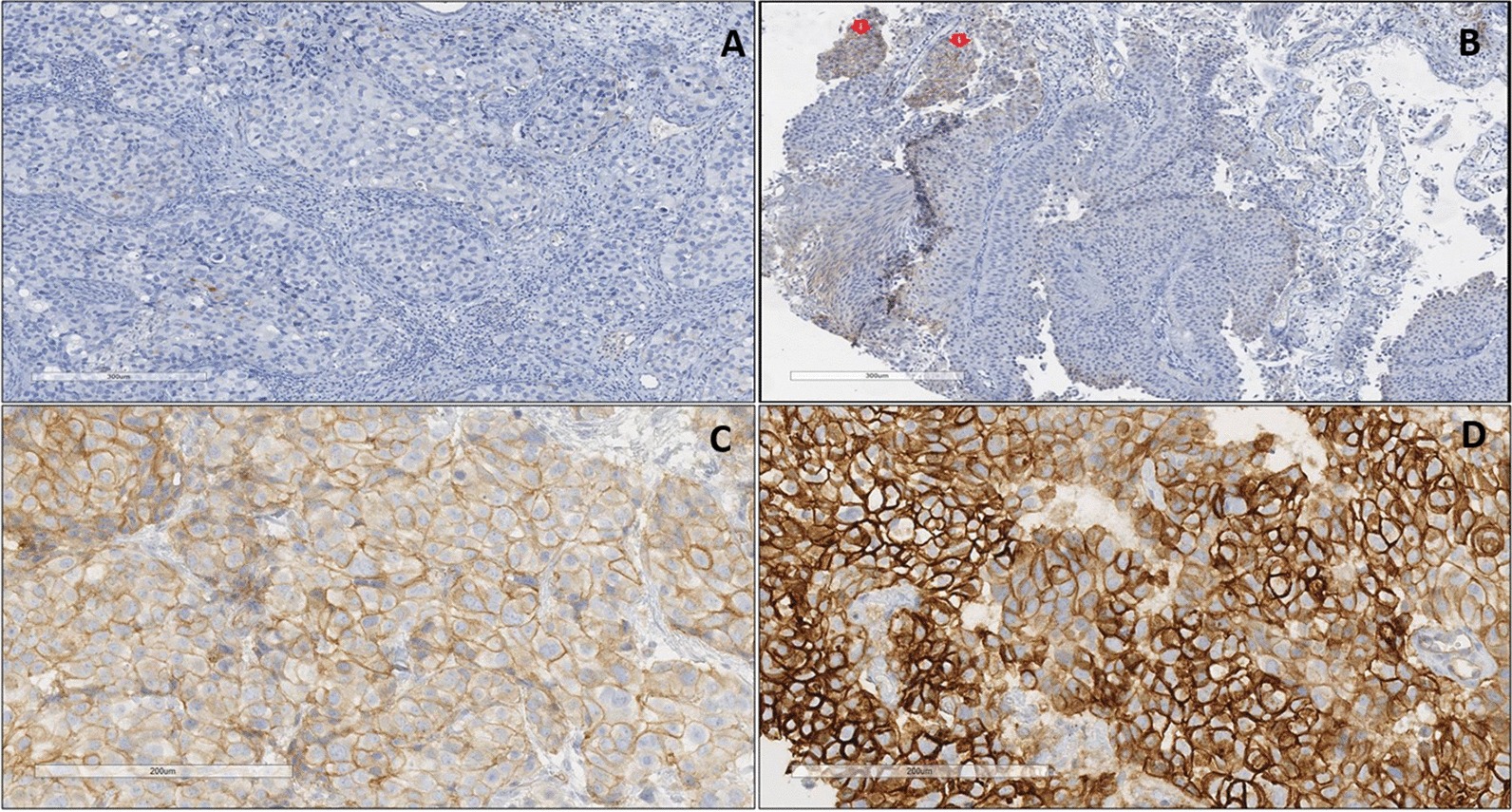
Anti-thrombomodulin IHC staining in BC tissues. **a** Negative: No membranous staining in < 5% of Bladder tumours **b** Positive (+): Positive membranous staining in up to 20% of Bladder tumours. Note the weak positive membranous staining (red arrows). **c** Positive (++): Positive membranous staining in up to 20–50% of Bladder tumours. Note the Moderate colour intensity. **d** Positive (+++): Positive membranous staining in > 50% of Bladder tumours. Note the high colour intensity. DAB detection. ×40 magnification

The anti-thrombomodulin primary monoclonal antibody used in this study shows membranous staining patterns in BC tissue sections (Fig. 2). Positive membranous staining was reported based on colour intensity and percentage of cells that were expressed. Generally, there was a significantly reduced thrombomodulin in Bladder tumours, compared to normal underlying tissues (X^2^(3) = 11.29, *p* < 0.010), as determined by the Chi-square test.

With respect cancer grading and staging, there was no significant association between patients’ cancer grade and Thrombomodulin (X^2^(2) = 0.380, *p* = 0.83), as determined by the Kruskal–Wallis test. There was however a significant association between patient’s cancer stage and Thrombomodulin (X^2^(2) = 6.50, *p* = 0.039), as determined by the Kruskal–Wallis test.

### HER-2/neu tissue expression in BC patients

**Fig. 3:**
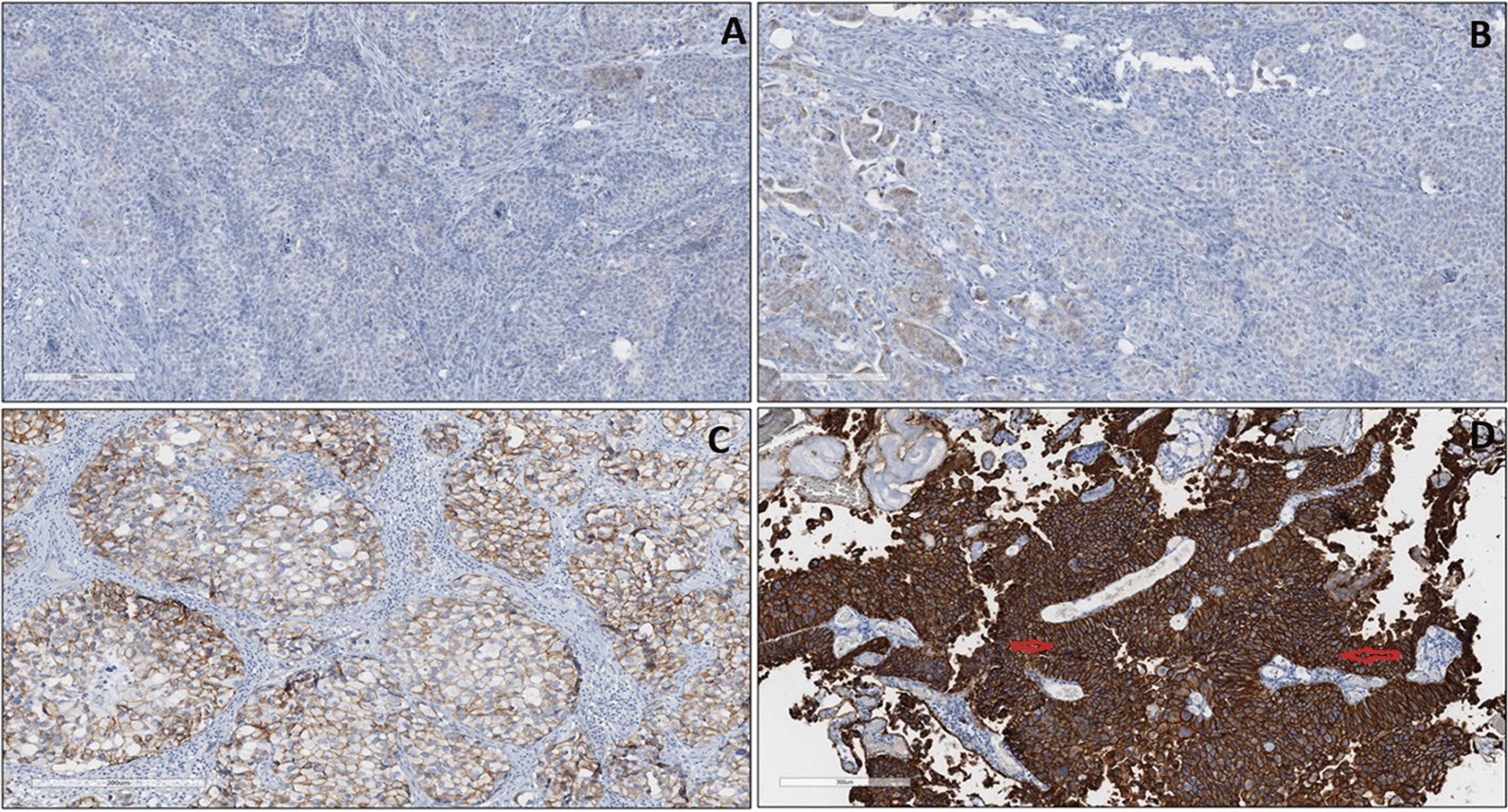
Anti-HER-2/neu antibody staining in BC tissues. **a** Negative: No staining or weak membranous staining in < 5% of BC tissues. **b** Positive (+): Weak intensity membranous staining in up to 20% BC tumours. **c** Positive (++): Moderate intensity membranous staining in 20–50% of BC tissues. **d** Positive (+++): Strong intensity membranous staining in > 50% of BC tissues (Brown colour, red arrows). DAB detection. ×40 magnification

The anti-HER-2/neu primary monoclonal antibody used in this study shows membranous staining patterns in BC tissue sections (Fig. 3). Positive membranous staining was reported based on colour intensity and percentage of cells staining positive. Generally, there was a significantly higher HER-2/neu expression in Bladder tumours, compared to normal underlying tissues (X^2^(3) = 8.82, *p* < 0.032), as determined by the Chi-square test.

With respect cancer grading, there was a significant association between patients’ cancer grade and HER-2/neu (X^2^(2) = 11.407, *p* = 0.003), as determined by the Kruskal–Wallis test. Further post-hoc testing using the Mann–Whitney test revealed significant difference between grade 1 and grade 2 (Z = -3.042, *p* = 0.002, r = 0.673) and a significant difference between grade 1 and grade 3 (Z = -2.843, *p* = 0.004, r = 0.606), both with a high Eta^2^ effect size. There was also a significant association between patient’s cancer stage and HER-2/neu (X^2^(2) = 16.092, *p* < 0.001), as determined by the Kruskal–Wallis test.

### S100P tissue expression in BC patients

**Fig. 4:**
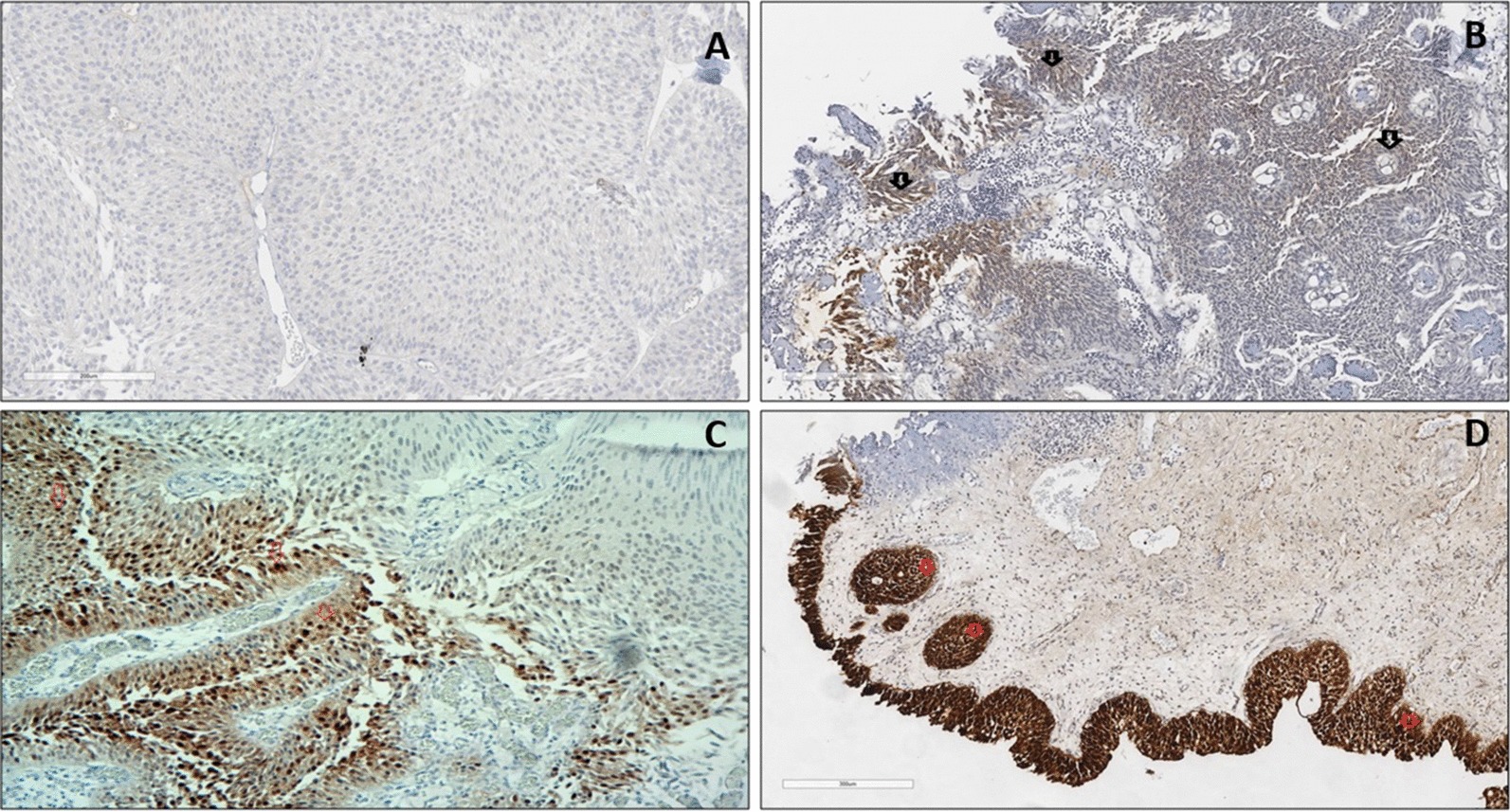
Anti-S100P antibody staining in BC tissues. **a** Negative: No staining or positive cytoplasmic/nuclear staining in < 5% of Bladder tumours. **b** Positive (+): Weak Nuclear/cytoplasmic staining in up to 20% of Bladder tumours (black arrows). **c** Positive (++): Moderate/strong positive nuclear and cytoplasmic staining 20–50% of Bladder tumours. **d** Positive (+++): Strong positive nuclear and cytoplasmic staining in > 50% of Bladder tumours. DAB detection. X40 magnification

The anti-S100p primary monoclonal antibody used in this study shows cytoplasmic and nuclear staining patterns in BC tissue sections (Fig. 4). Generally, there was a significant increase in S100P expression in Bladder tumours compared to those in normal tissues (X2(3) = 41.686, *p* < 0.001), as determined by the Chi-square test.

With respect to cancer grading, there was no significant association between patients’ cancer grade and anti-S100P expression (X^2^(2) = 0.206, *p* = 0.902), as determined by the Kruskal–Wallis. There was also no significant association between patient’s cancer stage and S100P (X^2^(2) = 2.134, *p* = 0.344), as determined by the Kruskal–Wallis test.

### COX-2 tissue expression in BC patients

**Fig. 5:**
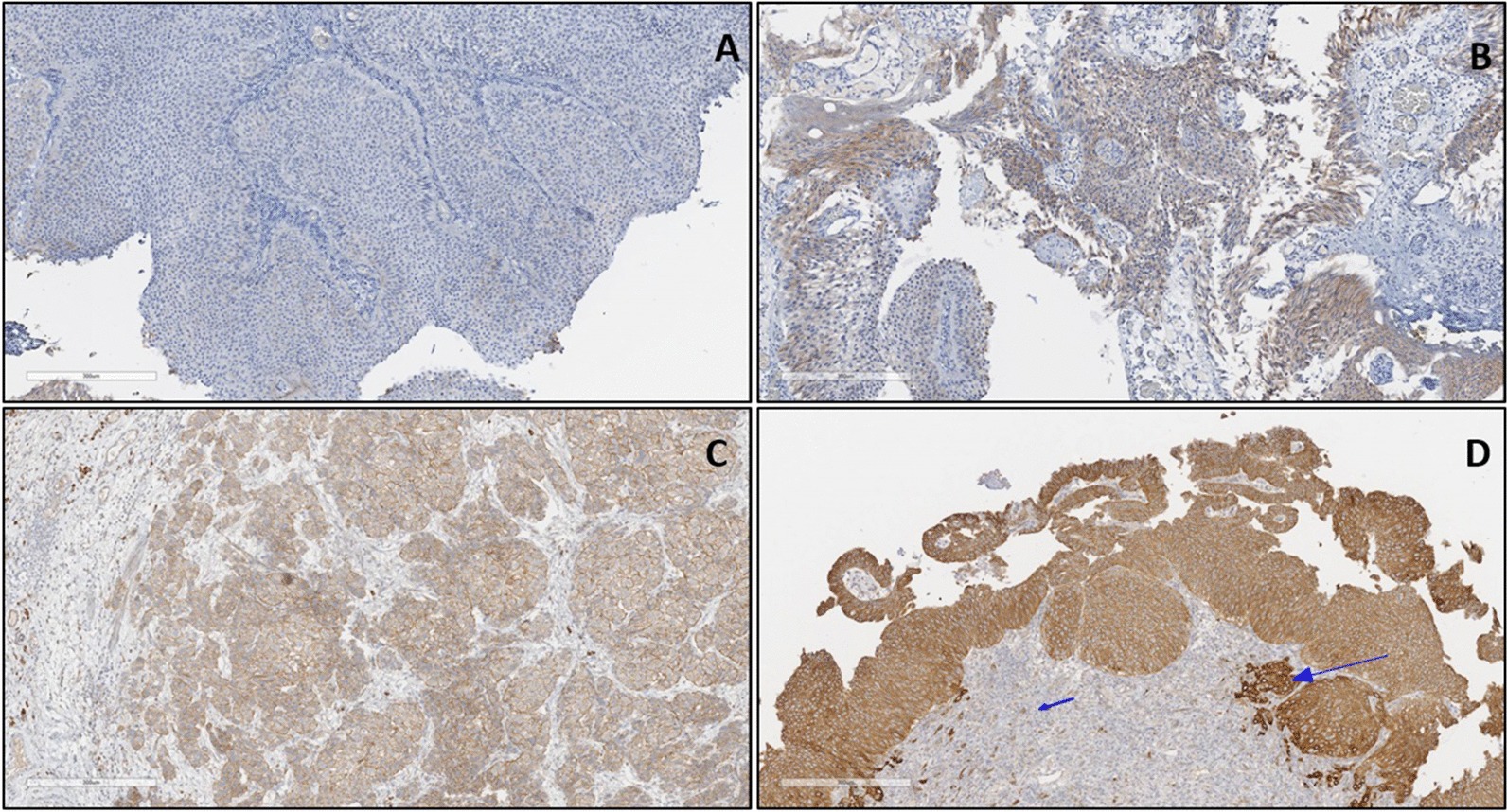
Anti-COX-2 antibody staining in BC tissues. **a** Negative: No staining/Positive cytoplasmic/membranous staining in < 5% of Bladder tumours. **b** Positive (+): Weak positive cytoplasmic/membranous staining in up to 20% of Bladder tumour cells (Brown colour). **c** Positive (++): Moderate/high positive cytoplasmic/membranous staining (brown colour) in in up to 20–50% of Bladder tumours. **d** Positive (+++): Strong positive cytoplasmic/membranous staining in in up to > 50% of Bladder tumours (bigger arrow). Note the absence of staining in underlying tissue (smaller arrow). DAB. ×40 magnification

The anti-COX-2 primary monoclonal antibody used in this study shows cytoplasmic and membranous staining patterns in BC tissue sections (Fig. 5). Generally, there was a significant increase in COX-2 expression in Bladder tumours, compared to those in normal tissues (X2(3) = 19.97, *p* < 0.001), as determined by the Chi-square test. With respect to cancer grading, there was no significant association between patients’ cancer grade and anti-COX-2 (X^2^(2) = 0.96, *p* = 0.61), as determined by the Kruskal–Wallis test. There was also no significant association between patient’s cancer stage and S100P (X^2^(2) = 1.65, *p* = 0.45), as determined by the Kruskal–Wallis test.

### Anti-CEACAM-1 tissue expression in BC patients

**Fig. 6:**
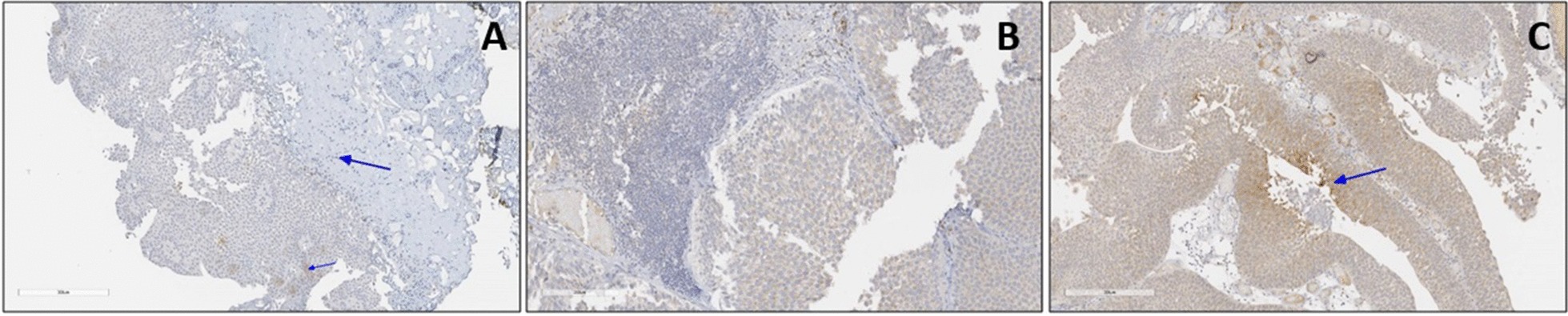
Anti-CEACAM-1 staining in BC tissues. **a** Negative: No staining/Positive membranous staining in < 5% of Bladder tumours. Small blue arrow shows weakly expressed bladder tumours. Big blue arrow shows underlying connective tissue. **b** Positive (+): Moderate membranous staining in up to 20% of Bladder tumours. **c** Positive (++): Positive membranous staining in 20–50% of Bladder tumour cells (Blue arrows). Ventana ultraview DAB detection. ×40 magnification

The anti-CEACAM-1 primary monoclonal antibody used in this study shows cytoplasmic/membranous staining patterns in BC tissue sections (Fig. 6). Generally, there was a significant change (loss of tissue expression) in CEACAM-1 in Bladder tumour, compared to normal tissues (X2(3) = 11.29, *p* < 0.001), as determined by the Chi-square test.

There was a significant association between patients’ cancer grade and CEACAM-1 expression (X^2^(2) = 6.19, *p* = 0.045), as determined by the Kruskal–Wallis. Further post-hoc testing using the Mann–Whitney test revealed significant difference between grade 1 and grade 3 (Z = -2.444, *p* = 0.015, r = 0.509). With respect to cancer staging, there was a significant association between patient’s cancer stage and CEACAM-1 expression (X^2^(2) = 10.78, *p* = 0.005), as determined by the Kruskal–Wallis test.

### Anti-VEGFR-3 tissue expression in BC patients

**Fig. 7:**
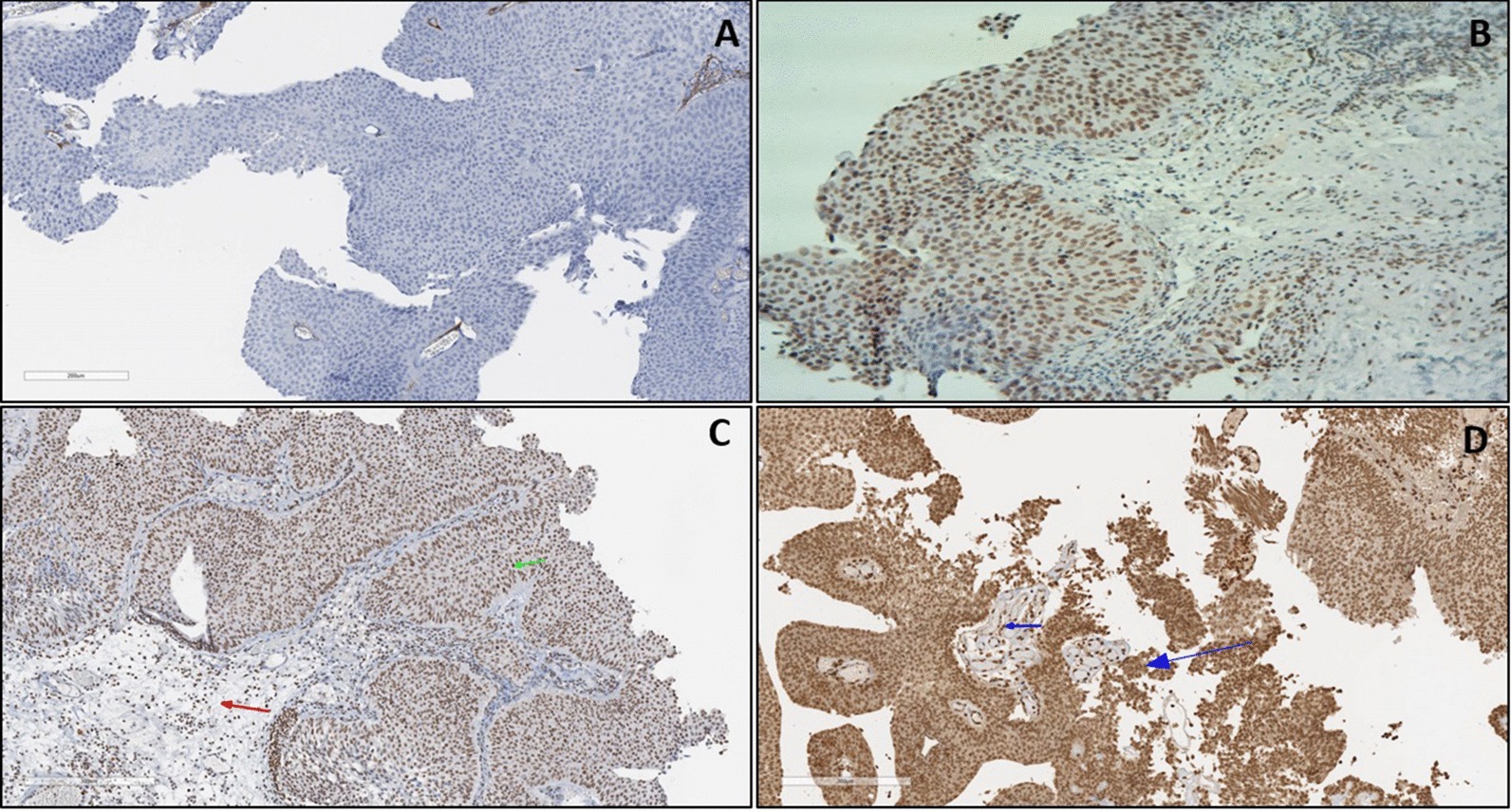
Anti-VEGFR3 staining in BC tissues. **a** Negative: No Staining or < 5% nuclear/cytoplasmic Staining. **b** Positive (+): Moderate intensity Nuclear/cytoplasmic staining in up to 25% of Bladder tumours **c** Positive (++) Moderate intensity nuclear/cytoplasmic staining in 25–50% of Bladder tumours (Green arrows). Nonspecific staining in underlying stroma cells (red arrows). **d** Positive (+++): Strong intensity nuclear/cytoplasmic staining in > 50% of Bladder tumour (big blue arrows). Smaller arrow shows nonspecific staining in stroma cells. Ventana Ultraview DAB. ×40 magnification

The anti-VEGFR-3 primary monoclonal antibody used in this study shows nuclear and cytoplasmic staining patterns in BC tissue sections (Fig. 7). Generally, there was a significant increase in VEGFR-3 expression in Bladder tumours, compared to normal tissues (X2(3) = 41.71, *p* < 0.001), as determined by the Chi-square test.

With respect to cancer grading, there was a significant association between patients’ cancer grade and VEGFR-3 (X^2^(2) = 4.18, *p* = 0.12), as determined by the Kruskal–Wallis. There was also no significant association between patient’s cancer stage and VEGFR-3 (X^2^(2) = 2.94, *p* = 0.23), as determined by the Kruskal–Wallis test. Table 2 summarises the results for antibody staining in bladder cancer patients.

**Table 2:** Summarised statistical results for antibody staining in BC

Antibody	Overall staining pattern in patients’ Bladder tumours (Chi square)	Association between antibody expression and cancer grade (Kruskal–Wallis test)	Association between antibody expression and cancer stage (Kruskal–Wallis test)	Association between antibody expression and cancer recurrence (Mann–Whitney test)
CD31	Increased expression in vascular cells (*p* < 0.001)	Not significant (*p* = 0.257)	Significant (*p* = 0.002)	Not significant (*p* = 0.69)
Thrombomodulin	Reduced/loss of expression (*p* < 0.010)	Not significant (*p* = 0.827)	Not significant (*p* = 0.039)	Not significant (*p* = 0.89)
HER-2	Increased expression (*p* < 0.032)	Significant (*p* = 0.003)	Significant (*p* < 0.001)	Not significant (*p* = 0.91)
S100P	Increased expression (*p* < 0.001)	Not significant (*p* = 0.902)	Not significant (*p* = 0.344)	Not significant (*p* = 0.23)
CEACAM-1	Reduced/loss of expression (*p* < 0.001)	Not significant (*p* = 0.045)	Significant (*p* = 0.005)	Not significant (*p* = 0.65)
COX-2	Increased expression (*p* < 0.001)	Not significant (*p* = 0.619)	Not significant (*p* = 0.439)	Not significant (*p* = 0.98)
VEGFR-3	Increased expression (*p* < 0.001)	Not significant (*p* = 0.123)	Not significant (*p* = 0.231)	Not significant (*p* = 0.38)

## Discussion

The main aim of this study was to evaluate the expression of various tissue-based biomarkers in BC patients and their association with cancer recurrence. This was done by assessing IHC staining in patient tissues following TURBT for the treatment of BC.

The results from this study showed a significant increase in anti-CD31 in vascular endothelial cells located in bladder tumour cells with some staining also located close to the tumour cells. As a marker of angiogenesis, these findings potentially show the presence of increased angiogenesis within the Bladder tumours. The scoring system used in this research examines both staining intensity and percentage positivity in tumour cells and has been used by others [27].

Anti-CD31 patterns reported in this study complements results by others [28] although those author’s used a different patient cohort. Both studies however show that, CD31 is a good marker for measuring angiogenesis using IHC. Results from this present study disagree with others [29] as no association between CD31 and cancer grade was observed. This could be due to differences in cancer types and grading systems. Patients with cancer stage pTa presented with higher CD31 compared to other BC stages. These findings suggest that increased CD31 in low pTa tumours may have an association with angiogenesis.

In this study, loss of expression or reduced thrombomodulin was observed within bladder tumour cells compared to normal bladder cells. Due to its tumour suppressive properties [30], loss of thrombomodulin observed in this present study, could potentially cause increased tissue differentiation, metastasis and recurrence. Although the exact mechanisms are not fully understood, earlier studies show that, thrombomodulin maintains cell–cell interactions and also inhibits degradation of the ECM; important factors in cancer proliferation [31, 32]. Our findings therefore further complement these earlier studies by highlighting the loss of Thrombomodulin within BC cells. In terms of Bladder specific research however, our research provides new information on Thrombomodulin staining patterns in bladder tumours.

High stage BC was significantly associated with reduced thrombomodulin suggesting that there was reduced cell to cell interaction due to the absence of thrombomodulin. This observation is in agreement with an earlier study which reported that, decreased thrombomodulin was significantly correlated with high cancer stage, differentiation and 5 year survival [33]. In this current study, there was no association between thrombomodulin (at diagnosis) and cancer recurrence. This observation should however be further investigated using larger sample sizes and increasing the length of follow up.

Our results also showed reduced CEACAM-1 in Bladder tumour cells compared to other tissues. Due to its angiogenic properties, an increase in CECAM-1, was expected in BC patients before TURBT. Although increased tissue expression of CEACAM-1 has been reported in human cancers [34], our findings of decreased expression may further explain earlier reports of a dual role of CEACAM-1 in BC [35]. Furthermore, the two IHC reporting systems (traditional scoring system using stain intensity/percentage vrs MVD) produced different results and raises further issues with validation.

Some studies have associated CEACAM-1 with aggressive tumour behaviour (i.e. high grade, advanced stage, metastasis and survival) in different human cancers [36, 37]. In our study, we report a significant association between loss of CEACAM-1 and BC stage and grade. Our study further attempts to evaluate this association with cancer recurrence. In this regard, there was no association between patients’ CEACAM-1 (at diagnosis) and BC recurrence.

There was generally a significant increase in COX-2 in Bladder tumour cells, compared to those in normal tissues. These findings are in agreement with a recent study by others in colonic adenocarcinoma tissues [4]. The results suggest that tumour cells of epithelial origin express high levels of COX-2 probably due to chronic inflammation and may potentially be a good biomarker. Furthermore, high stage and high grade patients presented with high staining intensity and percentage of tumour cells [4]. These observations were also present in the current study and suggests that COX-2 in cancer tissues (especially staining intensity) should be further investigated in BC.

With regards to cancer stage, our results are in contrast with others [5] who reported significant associations between COX-2 and cancer stage. Although both endometrial carcinoma (EC) and BC are of epithelial origin, differences in sample size (183 EC vrs 35 BC), duration of follow up (5 yrs. for EC vrs 2 yrs. for BC) and different antibody clones, may explain the differences in results reported by both studies.

The relationship between antibody expression (at diagnosis) vs BC recurrence was not significant. Nonetheless, the expression patterns of COX-2 in BC, reported in this present study, should be investigated further using larger sample sizes and longer follow up periods. COX-2 seems to be epithelial cancer specific and may be a useful marker for monitoring and management of BC patients following TURBT.

Anti-S100P was investigated in this present study because its cellular expression may interact with growth factors and their receptors, leading to several effects such as tumour proliferation [9]. The results from this present study showed that S100P was significantly expressed in BC cells in comparison with normal Bladder tissue and suggests this protein may be a good marker for identification of BC. In a similar study, S100A8 (which belongs to the same family as S100P) was highly expressed in TCC tissues compared to normal cells [38]. In disagreement with that study however, there was no correlation between S100P expression and patients’ cancer grade and stage in this present study.

Although it can be appreciated that S100P will be higher in high grade tumours due to increased cell proliferation [39], that observation was not made in this present study and could potentially be due to low sample size. Furthermore, comparing S100P at diagnosis, with S100P at 2–5 years following treatment, may provide confirmatory information with regards to recurrence and long-term outcome measures.

With regards to recurrence however, findings disagree with other researchers [39] who observed that positive S100P expression was strongly associated with early recurrence in HCC patients. Difference in patient cohorts and sample sizes may explain these variations. The results from this study nonetheless provides new information with regards to S100P staining in bladder tumour cells as well as its association with cancer grade, stage and recurrence following treatment via TURBT.

In various human cancers, different HER-2 rates and patterns have been reported [40–42]. HER-2 expression was therefore investigated in this present study due to its links to cancer growth in various human cancers, especially its utility in breast cancer treatment and management.

Our findings suggest that HER-2 was significantly higher in bladder tumour cells, compared to normal tissues. As a protein involved in signal transduction leading to increased cell proliferation, the increased expression observed in this present study potentially implicates HER-2 in BC growth. These observations complement results by others [43] who reported higher HER-2 expression in colorectal cancer cells compared to normal controls and another group [44] who also reported higher HER-2 in breast cancer patients. The differences between the studies however is that, this present study provides detailed information with regards to antibody clone, scoring system and optimises IHC protocol to the Ventana IHC autostainer.

High grade (G3) patients presented with higher HER-2 expression compared with low grade (G1), in agreement with [45], although their observations were made in breast cancer patients. These findings nonetheless suggest that HER-2 results in higher rate of cell differentiation that potentially leads to increased risk of recurrence and metastasis in BC patients.

High stage tumours (pT1/pT2) also presented with high HER-2, which complement findings from a previous study [45] involving breast cancer. It can be appreciated that, increasing HER-2 will lead to increased activation of the so-called MAPK pathway, which explains the association with aggressive tumour behaviour.

There was also a significant increase in VEGFR-3 in bladder tumour cells, compared to those in normal tissues. In a previous study [4], VEGF in colonic adenocarcinoma showed intensely positive staining compared to normal control tissues. However, control tissues were moderately positive for VEGF indicating the potential likelihood of false positive reporting. In this present study, there was also non-specific VEGFR-3 staining within nuclei of some underlying muscle cells, lymphatic endothelial cells and basal tissue cells. This observation in this and other study highlights the unspecific nature of VEGF staining, and raises the issues with standardisation in reporting slides.

A limiting factor for this study is the relatively low sample size. Validation of the findings larger multicentre studies could provide clear evidence of a usable biomarker profile as an adjuvant to BC management.

Future studies may also investigate antibody tissue immunoreaction, serum expression and urine levels simultaneously in all patients. This could provide much clearer data to enhance the search for a usable biomarker profile.

## Conclusion

It is proposed that by allowing urological surgeons access to laboratory markers such as HER-2, Thrombomodulin, CEACAM-1 and CD31, potentially, in the future, these biomarkers may be used in addition to, or in combination with, currently used scoring systems to predict cancer recurrence and progression.

Ultimately, our research findings may allow current NHS post-operative care protocols to be revised, improved and implemented within 6–8 years, although larger studies are needed in this area of research in order to make an impact on a national scale, and to promote good practice in the field of urology, pertaining to BC.

## Data Availability

The datasets used and/or analysed during the current study available from the corresponding author on reasonable request.

## Abbreviations

BC: Bladder cancer
COX-2: Cyclooxygenase-2
DAB: Diaminobenzidine
FFPE: Formalin-fixed paraffin-embedded
HER-2: Human epidermal growth factor-2
IHC: Immunohistochemistry
MAPK: Mitogen-activated protein kinase
NMIBC: Non-muscle invasive bladder cancer
TCC: Transitional cell carcinoma
TURBT: Transurethral resection of the bladder tumour
